# Consumer Ethicality Perception and Legitimacy: Competitive Advantages in COVID-19 Crisis

**DOI:** 10.1177/00027642211016515

**Published:** 2021-05-21

**Authors:** Gregory Payne, Alicia Blanco-González, Giorgia Miotto, Cristina del-Castillo

**Affiliations:** 1 Emerson College, Boston, MA, USA; 2 Universidad Rey Juan Carlos, Madrid, Spain; 3 Ramon Llull University, Barcelona, Catalonia, Spain

**Keywords:** ethics, brand ethicality perception, purchase intention, legitimacy, COVID-19, strategic management

## Abstract

The article aims to analyze the cause–effect relationship between Brand Ethicality Perception (CPE), legitimacy and purchase intention during the COVID-19 first wave, taking into consideration the mediation effect of the country of residence. Data collection was based on a survey launched during the COVID-19 lockdown in Madrid and New York. To analyze the established hypotheses and to test the multigroup analysis, we applied a structural modelling with SmartPLS. The research contributes to the field of brand management, and specifically of ethical branding, since it will analyze how stakeholders’ expectations fulfillment is key to build a consistent and valued brand meaning in crisis’ situations, demonstrating that ethical behaviors are key for gaining corporate legitimacy and, therefore, for improving business performances.

## Introduction

In 2020, the new SARS-like coronavirus, named COVID-19, swept across every national border, left health care systems collapsed, being compared with the World War of 1939–1945 in terms of its global impact on every household and business (Sneader & Singhal, 2020). Spain and the United States were ones of the most affected countries since the beginning of the pandemic (McKinsey, 2020). Between middle March and the end of May, Spain counts more than 236,000 infected people and more than 27,000 deaths. At least the 35% of these deaths were living in Madrid (Statista, 2020a). New York counted more than 206,000 infected persons and 17,500 confirmed death and this area was the most highly affected in the United States during that period (Statista, 2020b). People had to live in a lockdown in their houses, facing very difficult health, social, and economic situations. During the COVID-19 crisis, people’s lives changed abruptly and worldwide individuals experienced a decrease in the personal and family economic conditions, the lockdowns accelerated the adoption of digital adoption, which drove into new pattern consumption (McKinsey, 2020). According to several authors, COVID-19 changed everything and it looks like our lives will never be the same (Grigore et al., 2020). We are getting use to a “new normality,” where social distance, masks, no travelling, and extreme hygiene are compulsory (World Health Organization, 2020). Putting the world on an extended lockdown gave everyone the chance to reassess their priorities; to reevaluate the way we care for the sick and vulnerable; to reflect on our hectic, consumeristic lifestyles; and to consider if other more sustainable, democratic, and caring ways of conducting our affairs were possible (McQueen et al., 2020).

In this new and unexpected environment, companies changed their communication strategies and messages affecting customers’ perceptions (Xifra, 2020). According to the “Spring Update 2020” of the Edelman Trust Barometer (Edelman, 2020), people from all around the world declared that companies have the responsibility to help governments in the COVID-19 crisis’ resolution. Firms are expected to provide necessary products, to protect their employees’ well-being and financial interests, and to support their smaller suppliers. Companies are expected to behave ethically and work for the common good.

Consumer behavior has changed and more than the 65% of the population declared that, during the crisis, they supported brands that they trusted and say that the firms that prioritized only their economic benefits instead of people well-being will completely lose their consumers’ trust and loyalty (Edelman, 2020). According to this report, the number of people worldwide who decided to buy a new brand because they considered that this firm was more innovative and compassionate on managing the issues raised by the pandemic, increased on 7%. Besides, also increased the percentage of consumers that convinced other consumers to stop buying a brand which did not act properly in response of the pandemic.

We are living in a hyperconnected and transparent world, where consumers may easily access any kind of information and companies and brands are constantly under scrutiny (Castells, 2007). In this new environment, costumers “are increasingly demanding that their favorite brands behave ethically” (Iglesias et al., 2019) and corporate misconduct has negative effect on consumers’ perception toward brands and their reputation may be damaged forever at the eyes of the society moral judgement (Brunk, 2012). After misconduct associated with a brand, there is a negative impact on purchase intentions (Hsu et al., 2012; Mena et al., 2019). The brand choice is not just based on a brand’s functional and emotional benefits but also on customers’ identification with a brand’s ethical positions and views (Edelman, 2020; Kotler et al., 2012; Porter & Kramer, 2006). According to the institutional approach, brands have an ethical role that affect how the company itself is socially perceived by the community with which it interacts either directly or indirectly (Czinkota et al., 2014; Rindova et al., 2005).

When consumers develop a strong relationship with a brand, due to their association with a brand’s values, this link boosts recognition benefits from purchasing the brand’s products and thus increases brand equity (Iglesias et al., 2019). Besides, ethical brands acquire legitimacy because they fulfil stakeholders’ moral expectations since they align with their values and social norms (Deephouse et al., 2017). Legitimacy is an intangible asset which provide long-term and sustained competitive advantages for the firm (Bianchi et al., 2019; Czinkota et al., 2014; Miotto, del-Castillo, et al., 2020; Miotto et al., 2018) and influences purchase intention (Ozdora-Aksak et al., 2016). Legitimacy is granted when behaviors, values, and beliefs are shared with various stakeholders (Blanco-Gonzalez, Diéz-Martín, et al., 2020; Díez-Martín et al., 2010). To maintain legitimacy, companies need to respond to stakeholders’ different expectancies and engage in socially responsible and sustainable behaviors (Beddewela & Fairbrass, 2016; Blanco-González, Miotto, et al., 2020). Ethical brands represent firms that are able to communicate their commitment and positive impact on the society. During the COVID-19 crisis and afterward, these brands are perceived as taking care of people and planet, and not just of their own profit.

From April 15 to May 25, 2020, we performed a quantitative research, surveying more than 1,000 people living in Spain and the United States, with the objective of understanding the public perception on firms’ behavior and the grade of consumers’ acceptability and corporate legitimacy. The results show that people’s expectation on companies’ contribution during the COVID-19 crisis is high and their perception is different depending on the analyzed industry and company. A high percentage of respondent declared that, in future, they will support the ethical and legitimated brands more: The ones that, during the crisis, fulfilled their expectations and behave more ethically.

In this article, we specifically analyze and compare data form Madrid and New York citizens. Generally, people coming from different cultures have a different perception of brands ethicality, legitimacy, and purchase intention (Ford et al., 2005), therefore, for example, companies manage crisis situation adjusting to values and beliefs of the different countries (Bowen et al., 2018). The research objectives are focused on understanding the cause–effect relationship between Brand Ethicality Perception (CPE), legitimacy, and purchase intention during the COVID-19 first wave, taking into consideration the mediation effect of the country of residence.

Under this scenario, the research objectives of this article are focused on two main aspects. First, on understanding the cause–effect relationship between CPE, organizational legitimacy, and customers’ purchase intention during the COVID-19 first wave and lockdown, and, second, applying and analyzing the mediating effect of the country of residence. Besides, we also meant to highlight a shift in consumers’ paradigm during a worldwide health crisis that affected deeply and suddenly people lives. Furthermore, we meant to find a relationship with the context of extreme crisis and uncertainty such as the COVID-19 lockdown.

The research will contribute to the field of brand management, since it will analyze how stakeholders expectations fulfillment is key to build a consistent and valued brand meaning (Veloutsou & Guzman, 2017). The research will contribute to the field of ethical branding, demonstrating that ethical behaviors are key for gaining corporate legitimacy and, therefore, for improving business performances. Stating that the concept of CPE applied to ethical consumerism is a rather new field (Garanti, 2019) and it is worthy to be further and deeper analyzed in all its constructs and dimensions (Brunk & de Boer, 2020; Schamp et al., 2019), and particularly from the consumer decision-making process approach (Brunk, 2010). This article will contribute to better understand how brands ethicality may improve consumers purchase intention and, therefore, became a sustained competitive advantage.

The novelty of the project states on the recent disruptive changes that COVID-19 crisis created and the need of helping firms understand that a positive reaction based on ethical behavior will contribute to increase customers’ and stakeholders’ support and that only legitimate companies will survive to this crisis. Socially, demonstrating that ethical brands are the most successful ones will encourage managers to take decisions not just for the firms’ short-term economic profitability but also for the society’s common good.

The article is organized as follows: First, we describe a theoretical framework about the relationship between ethical brands, legitimacy, and purchase intention, then, we describe the applied methodology and results, and finally, we propose implications, conclusions, and future research lines.

## Ethical Brands, Legitimacy, and Purchase Intention

Companies are constantly assessed by public opinion and individuals scrutinize their actions employing a moral subjective filter putting them into the categories of right or wrong and good or bad, applying an ethical judgment to their behavior (Brunk, 2010). This increased consumers consciousness obliges many companies to introduce corporate social responsibility (CSR) as a strategic imperative (Schamp et al., 2019) and a built-in strategy (Carroll & Buchholtz, 2014). Aware of the lack of consensus in defining this term (Brunk, 2012; Carroll & Buchholtz, 2014; Crane et al., 2008; Garriga & Melé, 2004). We consider CSR as an umbrella concept that defines organizational management based on business ethics rules and principles (Carroll & Buchholtz, 2014; Crane et al., 2008; Dahlsrud, 2008; Matten & Crane, 2005; Salzmann et al., 2005; Strand, 2013). CSR relates to the decision-making process to assess, and maximize the positive impacts, while minimizing the negative ones, to all stakeholders from social, environmental, and economic perspectives (Carroll & Buchholtz, 2014; Miotto et al., 2018). In this definition of CSR, we include the ethical dimension of organizations, involving respect for stakeholders’ interests, human rights, and the environment, according to a global and long-term vision (Aguinis & Glavas, 2012; Carroll & Buchholtz, 2014; Crane et al., 2008).

The term ethics refers to a set of moral norms, principles, or values that guide people’s behavior (Sherwin, 1983). Moral philosophy applies two different approach to describe ethics: the deontology theory that considers a nonconsequentialist effect that guides evaluations and the teleology theory that represents a consequentialist approach to moral judgment (Brunk, 2012; Crane & Matten, 2007; Delgado-Alemany et al., 2020). According to the normative moral theory of deontology, inspired by the German philosopher Immanuel Kant, corporate ethics depends only from superior defined norms and rules, independently from the caused consequences of their effect (Clement, 2006). The teleological perspective evaluates not only the good or bad of an action by itself but its moral judgement depends on the effects and positive, negative, or neutral impacts that it may cause. In business ethics, the final assessment considers the trade-offs between increasing benefits and reducing harm for all parties affected (Crane & Matten, 2007). The deontological approach is focused on the origin of the individual behavior, the teleological one is focused on the social impact on the society (Brunk, 2012; Delgado-Alemany et al., 2020). An individual’s moral judgments may be a mix of both deontological norms and rules and teleological considerations of effects and impacts (Wang et al., 2016).

Therefore, ethics is the pillar of the responsible and sustainable corporate management and governance. Ethics is the guiding principle of CSR and it reflects on the brand image, reputation, and perception (Miotto & Youn, 2020). According to (Brunk, 2012, p. 551): “How un/ethical a company is perceived in conducting its business is inherently linked to its overall reputation and its ability to stay competitive in the marketplace.” Consumers’ subjective beliefs and un/ethical perceptions act as sources of attitude formation and they influence consumers’ purchase intentions (Das et al., 2019). In this line, we introduce the concept of ethical brands as the ones those behave with integrity, accountability, responsibility, and respect toward stakeholders (Iglesias et al., 2019). Ethical brands provide products and services characterized by a system of production, exchange, and management that respect providers, producers, communities, consumers, and the environment (Miotto & Youn, 2020). This system is based in economic profitability, people-to-people connections, social justice, and environmental sustainability (Szmigin et al., 2007).

Ethical brands engage in corporate sustainability practices, support stakeholder’s interests, and provide a competitive advantage (Bianchi et al., 2019; Blanco-Gonzalez, Diéz-Martín, et al., 2020; Iglesias et al., 2019). The integrity and ethicality of these brands fulfill stakeholders’ ethical expectations (Porter & Kramer, 2011), meet society’s moral values (Garanti, 2019), satisfy the consumer’s need for self-identity and self-expression (Das et al., 2019), build positive brand image and equity (Iglesias et al., 2019), lead to positive feelings and emotions toward the company (Garanti, 2019), enhance consumers’ trust (Swaen & Chumpitaz, 2008), improve financial performance (Luo & Bhattacharya, 2006), improve quality perception (Chernev & Blair, 2015), increase purchase intention (Bianchi et al., 2019; Szmigin et al., 2007), and decrease the possibility to switch to another unethical option (Phung et al., 2019). Ethical brands consider that, if they make decisions which are good for the public, these are going to be good for the firm as well (Romani et al., 2016), since they will be the ethical alternative which will fulfil the ethical, personal, and individual choice (Garanti, 2019). Companies actively seek to link their products to ethical attributes to improve their competitive position, build brand equity, or directly drive more sales (Schamp et al., 2019). Construing a firm as a responsible and ethical brand provides increased awareness, satisfaction, trust, and loyalty (Ajina et al., 2020).

Actually, consumers demand for ethically produced and sold products and services is at rise, converting ethical purchase in an important trend (Garanti, 2019; Govind et al., 2017; Schamp et al., 2019). By making an ethical purchase decision, consumers identify themselves and project their altruistic and positive biosphere value orientation (Yoganathan et al., 2019). People are moved to a moral obligation to help and this compelling reason to act foster ethical consumerism as a materialization of their altruistic and ethical orientation (Andersch et al., 2019). When purchasing ethical brands, consumers take an active role in shaping a better world and becoming responsible and sustainable consumers (Fuentes & Sörum, 2019). Nevertheless, it must be said that ethical purchase intention does not always convert into actual brand choice and purchase (Miotto & Youn, 2020). Even if several studies demonstrate that CPE affects the purchase intention positively (Bezençon & Etemad-Sajadi, 2015), there is still a gap in understanding the relationship between the ethical brand perception and the positive purchase behavior (Carrington et al., 2016; Longo et al., 2019; Szmigin et al., 2007). Brand familiarity and an actual good reputation increase the option to purchase intention when looking for an ethical alternative (Schamp et al., 2019). Due to the negativity bias, negative information is much more powerful than the positive one, especially because mainstream media and third-party social media easily expose more companies’ misconduct and negative evidence than their CSR practices (Schamp et al., 2019).

Brunk (2010) defines the six domains of CPE that can influence ethical perceptions of a company or brand and, therefore, the purchase intention are as follows: consumer (pricing, labelling, or advertising); employees (labor right respect, discrimination, or health); environment (pollution, recycling, or animal protection); local community and economy (positive and negative impacts of business and production processes in the nearby community); business community (accounting and reporting, competitive market rules, or corruption); and overseas community (exploitation of labor and natural resources, human rights, or relationship with local governments).

According to the theory of planned behavior (Ajzen, 1991) and the general theory of marketing ethics (Hunt & Vitell, 1986) information about the ethicality of brands and companies influence consumers attitudes and judgment, including the purchase intention (Govind et al., 2017). Ethicality of the brand can relate to a stakeholder-focused strategy, and the positive relationship between ethicality of a brand and brand trust, can result in increasing brand sales and performance (Mena et al., 2019). Considering the performed literature review about the relationship between ethics and consumer behavior, the following hypothesis is proposed:

**Hypothesis 1:** Consumer perceived ethicality positively and significantly affects the consumers purchase intention.

According to (Suchman, 1995, p. 574), corporate legitimacy is “a generalized perception or assumption that the actions of an entity are desirable, proper, or appropriate within some socially constructive system of norms, values, beliefs and definitions.” Corporate survival is significantly improved by demonstrations of conformity to the norms and social expectations within which the corporation operates (Ashrafi et al., 2020; Díez-Martín et al., 2021; Maignan & Ferrell, 2004). Through the fulfillment of stakeholders needs, organizations acquire legitimacy which provides them with an easier and more sustained access to the necessary resources to survive (Díez-Martín et al., 2013; Díez-Martín et al., 2020).

Legitimacy provides sustained competitive advantages which increase their options to grow and to improve future performance (Li et al., 2016; Miotto, del-Castillo, et al., 2020). Companies need to respond to stakeholders’ expectancies and engage in socially responsible and sustainable behaviors, explicitly expressing their ethical behavior (Beddewela & Fairbrass, 2016; Lamberti & Lettieri, 2011). Ethical brands are considered legitimated since they behave with integrity, accountability, responsibility, and respect toward all the stakeholders (Ajina et al., 2020; Iglesias et al., 2019). According to these arguments, we define the next hypothesis:

**Hypothesis 2:** Consumer perceived ethicality positively and significantly affects the corporate legitimacy.

Legitimacy of ethical brands increases purchase intention in several industries (Guo et al., 2014) and, especially, when the perception of ethicality is based on trust and transparency and when the mutual benefits are explicit (Miotto & Youn, 2020). The positive perception of the brand’s value and ethical behavior improve legitimacy and, therefore, the purchase intention (Lee & Shin, 2010). If value creation is not perceived as mutually beneficial for all stakeholders, legitimacy is not guaranteed and the competitive advantage is not sustained (Freudenreich et al., 2020). Moreover, legitimacy is directly related with the organizations’ capability to fulfill customer expectations and values (Díez-Martín et al., 2021). It is achieved when an organization performs based on moral or ethical values which overlap with its stakeholders expectancies (Díez-Martín et al., 2021). Based on these statements, we propose this hypothesis:

**Hypothesis 3:** Corporate legitimacy positively and significantly affects the consumers purchase intention.

Cultural and social context influences individuals’ values, attitudes, and behavior (Bowditch et al., 2007). National culture has a relevant role in defining cultural values and determining ethical attitudes (Christie et al., 2003) since perception about what is wrong or right depends on the cultural and environmental context of each region (Adler & Harzing, 2009). Ferrell and Gresham (1985) considered that individuals’ decision-making process is influenced by inherent factors such as nationality. Thus, differences in the perceptions of ethics as well as in behavioral patterns can be identified between people from different countries (Hood & Logsdon, 2002). In fact, many authors have explored the effect of cultural factors on ethics’ perceptions, highlighting differences between the United States and Spain in the perception of ethical values (Alas, 2006).

In the past few months, several studies have been performed to understand how the different countries were affected economically and socially by the pandemic and there is a consensus about that there was an homogeneous reaction between the citizens of the Western Countries since policy makers adopted very similar measures such as lockdowns, economic aids, shop and restaurants opening restrictions, and so on (Kraus et al., 2020). Nevertheless, we could not find specific research about the relationship between CPE, legitimacy, and purchase intention during the COVID-19 crisis in different countries.

Therefore, to test this comparison, we proposed the following hypothesis:

**Hypothesis 4:** The country of origin moderates the relationship between consumer perceived ethicality and consumer purchase intention.
**Hypothesis 5:** The country of origin moderates the relationship between consumer perceived ethicality and corporate legitimacy.
**Hypothesis 6:** The country of origin moderates the relationship between legitimacy and consumer purchase intention.

**Figure 1.**
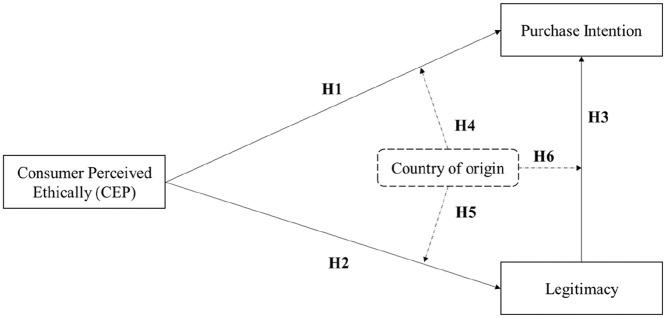
Proposed model. *Source*. Own elaboration.

To better clarify the research model that relates the three constructs of CPE, legitimacy, and purchase intention, we design the model showed in Figure 1.

## Sample and Methodology

### Research Setting and Data Collection

The considered research setting for this analysis were New York City (the United States) and Madrid (Spain). These two cities have in common several conditions that we considered very important as a sample to make a comparison between two different environments as the American and the Spanish. Both cities experienced a strict lockdown between March 2020 and June 2020 to decrease the spread of the COVID-19 virus (Mervosh et al., 2020). Both cities were the most affected in their respective countries in terms of number of infected people and death. According to the U.S. Department of Health, between March and June, New York counted more than 206,000 infected persons and 17,500 confirmed death and this area was the most highly affected in the United States during that period (Statista, 2020b). At the same time, Spain counted more than 236,000 infected people and more than 27,000 deaths, where at least the 35% of them were living in Madrid area (Statista, 2020a). Therefore, these two cities were selected since they suffered similar consequences due to the pandemic, being respectively the focus of the contagious in their countries. Besides, these two cities are the economic capital of their countries, having both a high population density and daily concentration of people circulation. In both cities, the governments applied measures to try to reduce the impact of the pandemic which had relevant social and economic consequences, however, the type of policies applied differ and affected citizens as well as companies in a diverse manner. Therefore, we considered interesting to compare the citizens’ perceptions on the role that companies had during this crisis as well as to analyze how their legitimacy levels have changed during this period and how it affected the ethicality perception and the purchase intention.

The data collection was based on a survey that included several questions regarding companies’ responsible behavior during the COVID-19 lockdown, the impact on citizens’ future consumption habits and the legitimacy perception in Madrid and New York. The number of effective responses were 379 for Madrid and 650 for New York (Table 1). For the Madrid data collection, a survey was sent directly to a database of contacts, while for New York data was collected from the Amazon Mechanical Turk (MTurk) online panel. Amazon MTurk is a crowdsourcing market to facilitate data collection, where researchers place their projects and anonymous workers participate in projects for monetary incentives (Kees et al., 2017). MTurk is a web-based data source that allows researchers to recruit demographically diverse subjects and collect good-quality data (Kees et al., 2017; Paolacci & Chandler, 2014). During the COVID-19 pandemic, MTurk provided the opportunity to collect a large nationwide sample in a relatively short amount of time, facilitating timely examination of the initial impact of the COVID-19 pandemic in the United States (Tull et al., 2020).

**Table 1.** Technical Specifications of the Study.

Population universe	Spanish and American citizens
Sampling technique	Probabilistic standardized by population and regional structure
Method of collecting information	Auto-administered online surveys
Person surveyed	Resident population in Madrid and the state of New York with age more than 18 years
Sample size	Madrid = 379 • Age: 18-25 = 30.9%; 26-35 = 26.3%; 35-45 = 18.8%, 46-55 = 20.9%; >56 = 3.1% • Gender: female = 50.6%; male = 49.4% • Profession: student = 27.8%; employed = 58.4%; unemployed = 7.8, other = 5.9%
	New York = 650 • Age: 18-25 = 20.09%; 26-35 = 38.9%; 35-45 = 20.8%, 46-55 = 16.9%; >56 = 3.3% • Gender: female = 47.6%; male = 52%; other = 0.4% • Profession: student = 13%; employed = 66.1%; unemployed = 16.1, other = 12.4%
Dates of information collection	From the April 17 to the May 30, 2020

### Measurement and Methodology

The considered constructs were measured through adapted items from existing scales as well as from the content of previously developed research on the field (Table 2). Besides, we customized some questions adapting them to the COVID-19 lockdown context, taking into consideration the conclusions of the “Spring Update 2020” of the Edelman Trust Barometer (Edelman, 2020) which specified that the main consumers’ concern around the companies ethical responsibilities during the lockdown were focused on companies working for solving the health global crisis, companies protecting the health of their employees, companies taking care not just about their profit but working for the common good, and the society well-being. Respondents were asked to reply considering their consumer habits.

**Table 2.** Measurement Instrument.

Factor	Item	Description
Brand ethicality perception	ETHIC01	Are they fulfilling the health requirements?
	ETHIC02	Are they an example of how companies should be behaving in other countries under this situation?
	ETHIC03	Are they honest?
	ETHIC04	Are they responsible with their actions?
	ETHIC05	Do their actions represent benefits for society?
	ETHIC06	Considering the health emergency. Are they fulfilling the law?
	ETHIC07	Are they helping their employees to fulfill the law?
Legitimacy	LEG01	In general terms . . . is your perception regarding companies and their role in this crisis acceptable and appropriate?
Purchase intention	INTENC01	Will your perceptions about companies’ behavior during this crisis affect your purchase decisions in the case of those companies that have not behaved properly?
	INTENC02	Will the origin of products affect your purchase decisions? For example, made in China, made in Bangladesh, made in Spain

We applied a 5-point Likert-type scale for their measurement in the survey with 0 referring to *strongly disagree* and 5 referring to *strongly agree*. To measure CPE, we used an adapted scale considering the research carried out by several authors and tested in previous research (Brunk, 2012; Brunk & de Boer, 2020). To measure legitimacy, we considered the work developed by several authors (Alexiou & Wiggins, 2019; Blanco-Gonzalez, Miotto, et al., 2020; Chung et al., 2016; Deephouse et al., 2017; Miotto, del-Castillo, et al., 2020). Finally, regarding purchase intention, we were inspired by the research performed by Hsu et al. (2012) and Phung et al. (2019).

To analyze the established hypotheses and to test the multigroup analysis (MGA), we applied a structural modelling with SmartPLS. This technique was chosen because it is a strong method of analysis (Chin et al., 2003) that offers adequate advantages to develop this research (Sarstedt et al., 2011) since this technique supports MGA (Hair, Sarstedt, et al., 2017; Henseler et al., 2016; Sarstedt et al., 2011). Following (Hair, Hult, et al., 2017), we considered that the use of partial least squares structural equation modeling (PLS-SEM) was more suitable than the CB-SEM for this research because PLS-SEM is mostly used for predictive causal analysis, where the explored issues are complex and the existing theoretical knowledge about them is relatively scarce (Hair et al., 2014). CB-SEM is a methodology more suitable when the research objective is theory testing, theory confirmation, or comparison of alternative theories. Furthermore, in the proposed research model, reflective and formative constructs were considered, therefore, PLS-SEM was the best tool option. The samples of 650 and 379 considered for our analysis are appropriate since previous studies have identified a sampling threshold for PLS-SEM of 100 subjects (Reinartz et al., 2009).

## Data Analysis and Results

### Descriptive Analysis

We carried out a descriptive analysis to understand the values of the considered variables measuring citizens’ perceptions on the brands’ ethical behavior, legitimacy, and purchase intention (Table 3). The results show the different factors and the corresponding items with their mean and standard deviation obtained through the analysis of the data collected from citizens of Madrid and New York.

**Table 3.** Descriptive Analysis.

Factor	Item	Madrid (Spain)			New York (The United States)		
		Mean	Standard deviation	Average factor value	Mean	Standard deviation	Average factor value
Brand ethicality perception	ETHIC01	3.325	1.136	3.279	3.394	1.062	3.374
	ETHIC02	2.987	1.269		3.252	1.118	
	ETHIC03	3.098	1.211		2.984	1.186	
	ETHIC04	3.253	1.206		3.497	1.157	
	ETHIC05	3.367	1.226		3.451	1.052	
	ETHIC06	3.567	1.199		3.647	1.040	
	ETHIC07	3.359	1.182		3.391	1.085	
Legitimacy	LEG01	3.182	1.205	3.182	3.368	1.058	3.368
Purchase intention	INTENC01	3.641	1.278	3.545	3.700	1.101	3.398
	INTENC02	3.449	1.525		3.096	1.432	

The results indicate that the average value of the considered variables (brand ethicality perception, legitimacy, and purchase intention) is relatively similar in Madrid and New York. In Madrid, the average value of brand ethicality perception is 3.3, for legitimacy 3.2, and for purchase intention 3.5 over 5. In New York, the results are 3.4 for brand ethicality perception, legitimacy, and purchase intention over 5.

### Assessment of Measurement Model and Invariance Measurement Across Groups

The reliability and validity of the measurement model was tested and is presented in Table 4. For the reflective items forming ethical brand, all the Cronbach’s alphas are presented, and they meet the required values of 0.70 (Nunnally & Bernstein, 1994). The composite reliability results are appropriate since they are all over 0.60 (Bagozzi & Yi, 1988). When considering the average variance extracted values, over 0.50 are considered acceptable (Fornell & Larcker, 1981). Furthermore, the standardized loadings of the reflective items are presented as well as their significant value (*p* < .01) which shows that they were meaningfully linked to their respective variable. Regarding the formative variable of purchase intention, the collinearity (variance inflation factor [VIF]) value indicates every item is under the correct level of VIF < 5 (Hair et al., 2014). The standardized weights are shown as their significant values (*p* < .01), which indicates that one of purchase intention’s formative item is significant, while the other is not, having a *t* value under 2 for both Madrid and New York. However, since the loading of this item was high (over 0.50), it was maintained as valid (Hair et al., 2014).

**Table 4.** Measurement Model Reliability and Validity.

Factor	Item	Weights/loadings	*t*	VIF	CA	CR	AVE
*Madrid*							
CPE	ETHIC01	0.789	31.535		0.917	0.934	0.669
	ETHIC02	0.864	55.593				
	ETHIC03	0.848	44.804				
	ETHIC04	0.875	49.537				
	ETHIC05	0.730	20.628				
	ETHIC06	0.772	30.993				
	ETHIC07	0.837	45.620				
Legitimacy	LEG01	1.000			1.000	1.000	1.000
Purchase intention	INTENC01	0.010	0.036	1.279			
	INTENC02	0.535	2.349	1.207			
*New York*							
CPE	ETHIC01	0.811	48.021		0.894	0.917	0.613
	ETHIC02	0.810	46.384				
	ETHIC03	0.822	56.914				
	ETHIC04	0.722	24.842				
	ETHIC05	0.734	29.685				
	ETHIC06	0.745	32.184				
	ETHIC07	0.827	54.062				
Legitimacy	LEG01	1.000			1.000	1.000	1.000
Purchase intention	INTENC01	0.170	0.635	1.409			
	INTENC02	0.949	7.841	1.072			

*Note*. VIF = variance inflation factor; AVE = average variance extracted; CPE = brand ethicality perception; CA = Cronbach’s alpha; CR = composite reliability.

Table 5 shows the results regarding the discriminant validity applying the heterotrait–monotrait ratio of correlations method which presents that every ratio was lower than 0.85 (Henseler et al., 2015). Thanks to all these fulfilled prerequirements, we consider that the model is accepted. We concluded that the proposed model offers appropriate evidence of reliability, convergent, and discriminant validity (Table 5) for the reflective constructs as well as in terms of collinearity and weight-loading relationship and significant levels for the formative construct.

**Table 5.** Discriminant Validity (Heterotrait–Monotrait Ratio of Correlations).

Factor	Legitimacy	
	Madrid	New York
Brand ethicality perception	.829	.778

To develop MGA and to compare the path coefficients between citizens’ perceptions in Madrid (Spain) and New York (USA), the acceptability of the models as well as the measurement invariance have to be evaluated (Hair et al., 2011; J. Henseler et al., 2015). To fulfill this requirement, we applied the measurement invariance of composite method proposed by Henseler et al. (2016) was applied. Measurement invariance of composite method includes three steps: (a) the configural invariance assessment, (b) the establishment of compositional invariance assessment, and (c) the assessment of equal means and variances. Table 6 shows partial measurement invariance for both groups, thus, the analysis of the MGA’s group differences using the results from PLS-SEM can be applied (Henseler et al., 2016).

**Table 6.** Results of Invariance Measurement Testing Using Permutation.

Constructs	Configural invariance	Compositional invariance		PMI	Equal mean assessment			Equal variance assessment			FMI
		C = 1	CI		Diff	CI	Equal	Diff	CI	Equal	
Brand ethicality perception	Yes	1.000	[1.00, 1.00]	Yes	–0.103	[–0.14, 0.13]	Yes	0.265	[–0.19, 0.16]	No	No
Legitimacy	Yes	1.000	[1.00, 1.00]	Yes	–0.166	[–0.14, 0.13]	No	0.261	[–0.16, 0.14]	No	No
Purchase intention	Yes	0.746	[0.69, 1.00]	Yes	0.046	[–0.13, 0.13]	Yes	0.186	[–0.15, 0.14]	No	No

*Note*. PMI = partial measurement invariance; FMI = full measurement invariance.

### Assessment of the Structural Model and Multigroup Analysis

The results confirm the proposed Hypotheses 1 and 2 showing the relationship between ethicality brand perception and legitimacy (Table 7). Nevertheless, Hypothesis 3 is not confirmed, since results show that, in this situation, legitimacy does not have a positive impact on the purchase intention.

**Table 7.** Hypotheses Testing.

Hypotheses	Madrid		New York	
	β	*t*	β	*t*
H1: CPE–Purchase intention	.248	2.010	.191	2.343
H2: CPE–Legitimacy	.797	32.987	.740	28.355
H3: Legitimacy—Purchase intention	.044	0.430	.055	0.596
Madrid: legitimacy *R*^2^ = .63, *Q*^2^ = 0.63, purchase intention *R*^2^ = .16, *Q*^2^ = 0.12; New York: legitimacy *R*^2^ = .55; *Q*^2^ = 0.54; purchase intention *R*^2^ = .16; *Q*^2^ = 0.12				

To develop the MGA, the parametric test was used (Hair, Hult, et al., 2017), where path coefficient differences lower than 0.05 represent significant differences between the considered groups. The Henseler’s boostrap-based MGA (Reinartz et al., 2009), and the Permutation test (Chin, 2010) were the nonparametric methods applied, since these two techniques are the most conservative ones for PLS-SEM to assess differences between groups. For the Henseler MGA method, a *p* value of differences between path coefﬁcients lower than .05 or higher than .95 indicates at the 5% level signiﬁcant differences between speciﬁc path coefﬁcients across two groups (Hair, Hult, et al., 2017; Reinartz et al., 2009), whereas for the permutation test differences are only at the 5% level signiﬁcant, when the *p* value is smaller than .05.

The three methods used for the MGA support the same conclusions. This consistence provides a multimethod confirmation of the obtained results (Table 8). The results demonstrate that no significant differences appear between the perceptions of the citizens in Madrid and New York when considering the relationships between the analyzed variables. Hypotheses 4, 5, and 6 are rejected.

**Table 8.** Hypotheses Testing MGA.

	*p*-Value differences				
Moderating effect hypotheses	Path coefficient difference	Parametric test	Henseler MGA	Permutation test	Supported
Hypothesis 4: CPE–Purchase intention	0.017	0.902	0.886	0.872	No, No, No
Hypothesis 5: CPE–Legitimacy	0.057	0.140	0.111	0.106	No, No, No
Hypothesis 6: Legitimacy–purchase intention	–0.011	0.937	0.927	0.929	No, No, No

*Note*. MGA = multigroup analysis; CPE = Brand Ethicality Perception.

## Discussion and Implications

During the COVID-19 crisis, people’s lives changed and the “new normal” drove into new pattern consumption (McKinsey, 2020). During the first wave of the pandemic, long months of lockdown gave people the chance to reassess their priorities, to reflect on our consumeristic habits, and to consider new and more sustainable and responsible ways of buying (Grigore et al., 2020). In this new and unexpected environment, companies changed their communication strategies and messages affecting customers’ perceptions on their ethical behavior (Xifra, 2020).

The results of our research confirm the theoretical literature review that states that, lately and also specifically during the lockdown, companies are expected to behave ethically and work for the common good (Edelman, 2020; Iglesias et al., 2019; Miotto & Youn, 2020). The results confirm that consumers’ demand for ethical brands is at rise, converting ethical purchase in an important trend (Garanti, 2019; Govind et al., 2017; Schamp et al., 2019). The ethical purchase decision is an act where consumers identify themselves and project their altruistic and positive biosphere value orientation (Yoganathan et al., 2019). Results confirm that a high ethicality in brand perception increases the brand purchase intention, since consumers take an active role in shaping a better world and becoming responsible and sustainable citizens (Fuentes & Sörum, 2019).

At the other side, results show that during the lockdown brands which were considered as ethical were also perceived as legitimate. These results confirm the theory that ethical brands enjoy a higher level of legitimacy, since they fulfill stakeholders demands, reflexing their values and having a positive impact on the society (Czinkota et al., 2014). On the contrary, results do not confirm the positive relationship between legitimacy and purchase intention. This discrepancy may be explained by the fact that legitimacy is an intangible asset which provide long-term and sustained competitive advantages, but not a quick change in the consumer behavior (Bianchi et al., 2019; Czinkota et al., 2014; Miotto, del-Castillo, et al., 2020). Legitimacy influences purchase intention (Ozdora-Aksak & Atakan-Duman, 2016), but its positive impact on the actual buying action may take several time before being explicit and tangible.

Research findings confirm a positive relationship between CPE, legitimacy, and purchase intention during the lockdown, with no relevant differences between the two analyzed cities. In both cases, the positive perception of the brand role in helping with the resolution of huge global issues related with the pandemic increases brand legitimacy and purchase intention. The homogeneous reactions of two different countries’ citizens confirms a recent research performed by several authors that concludes that during the COVID-19 pandemic, the economic and social impact and reactions were very similar in Western Countries due to the alignment of policy-makers decisions (lockdowns, economic aids, restrictions, etc.; Kraus et al., 2020).

Implications for brand management highlight that, since stakeholders expectations fulfillment is key to build a consistent and valued brand meaning (Veloutsou & Guzman, 2017), shape a brand based on people values increases legitimacy and, therefore, purchase intention. Firms that are managed ethically, in an environmentally sustainable and socially responsible manner are more likely to design brands that consumers are more willing to support. Consumers who buy ethical brands are responsible and sustainable, and this is confirmed in a situation of health crisis. As per Bezençon and Etemad-Sajadi (2015), this research demonstrates that CPE positively affects purchase intention. Moreover, it incorporates a variable of great interest in the academic field: organizational legitimacy (Czinkota et al., 2014; Deephouse et al., 2017; Lee et al., 2018; Li et al., 2016; Miotto & Youn, 2020). This research demonstrates the effect of ethics on legitimacy and that the impact of legitimacy on purchase intention is not immediate since legitimacy has a long-term impact on customer perception. The design of consistent and effective communication strategies is vital for companies that wants to be legitimated and improve their customer likability. Information sharing and accountability, for example, are key factors for achieving legitimacy and, in the long-term, improve brand performance (Miotto, del-Castillo, et al., 2020).

According to the literature review, previous research proved that CPE affects positively or negatively the brand perception according to the grade of perceived corporate ethical behavior (Brunk, 2012), it affects the approach to CSR-related aspects (Brunk & de Boer, 2020), the customer-brand performance such as purchase intentions and loyalty (Bianchi et al., 2019; Mena et al., 2019; Szmigin et al., 2007), the customer satisfaction and market value (Luo & Bhattacharya, 2006) and it defines increasing consumer habits based on ethical consumer-brand attributes and attitudes (Andersch et al., 2019; Carrington et al., 2016; Fuentes & Sörum, 2019; Govind et al., 2017; Yoganathan et al., 2019).

From the consumer decision-making process approach, this research helps better understand how brands ethicality improves consumers purchase intention and, therefore, became a sustained competitive advantage in a crisis situation where customers are immersed in a uncertain environment. According to the research results, since ethical behaviors are key for gaining corporate legitimacy and, therefore, for improving business performances, it is necessary to define communication strategies focused on messages that prove the high grade of brand ethicality, since ethical attributes are difficult to perceive (Schamp et al., 2019).

Managers should take into consideration the importance of communicating effectively and emotionally the positive moral attributes of a brands in order to be considered by the consumers in the screening phase of the purchase journey (Schamp et al., 2019). Accountability, information sharing, and good corporate governance and business ethics practices are key factors for ethical brands perception. CSR and environmental sustainability reports are effective tools for communicating the brands positive impact (López-Balboa et al., 2021). Brand managers should adopt transparent and accountable reporting practices that will support and act as a reason to believe of the brands communication strategies and messages.

The novelty of this project states on the recent disruptive changes that COVID-19 crisis created, such as the great downsize in the household spending (Baker et al., 2020) and the need of helping firms understand that a positive reaction based on ethical behavior will contribute to increase customers’ and stakeholders’ support and that only legitimate companies will survive to this crisis. Thinking about the future of branding in the new normality, managers should be aware that taking decisions, not just for the firms’ short-term economic profitability but also for the society common good, will improve brand legitimacy and consumer purchase intention.

### Limitations and Further Research

Regarding the limitations and future research lines of our study, we would like to point out that this article gathered the data on a very critical moment where citizens were highly affected by the sanitary crisis and the lockdown, therefore their responses might have been affected by their specific emotional state. A future investigation, through a second survey after few months from the lockdown, could help understand the real social impact that the COVID-19 crisis has caused in the medium and long term in the consumer habits and brands perception.

A follow-up research would contribute to the theory of planned behavior, analyzing if the COVID-19 crisis caused a real social change or just a short-term modification in brands perception. If confirmed, this social change would affect the corporate’s context and organizational practices such as marketing strategy, human resources management, and corporate governance.
